# Dynamic Mechanisms of Cell Rigidity Sensing: Insights from a Computational Model of Actomyosin Networks

**DOI:** 10.1371/journal.pone.0049174

**Published:** 2012-11-05

**Authors:** Carlos Borau, Taeyoon Kim, Tamara Bidone, José Manuel García-Aznar, Roger D. Kamm

**Affiliations:** 1 Aragón Institute of Engineering Research (I3A), Department of Mechanical Engineering, University of Zaragoza, Zaragoza, Spain; 2 Institute for Biophysical Dynamics, University of Chicago, Chicago, United States of America; 3 Departments of Biological and Mechanical Engineering, Massachusetts Institute of Technology, Cambridge, United States of America; 4 Department of Mechanics, Politecnico di Torino, Torino, Italy; Emory University/Georgia Insititute of Technology, United States of America

## Abstract

Cells modulate themselves in response to the surrounding environment like substrate elasticity, exhibiting structural reorganization driven by the contractility of cytoskeleton. The cytoskeleton is the scaffolding structure of eukaryotic cells, playing a central role in many mechanical and biological functions. It is composed of a network of actins, actin cross-linking proteins (ACPs), and molecular motors. The motors generate contractile forces by sliding couples of actin filaments in a polar fashion, and the contractile response of the cytoskeleton network is known to be modulated also by external stimuli, such as substrate stiffness. This implies an important role of actomyosin contractility in the cell mechano-sensing. However, how cells sense matrix stiffness via the contractility remains an open question. Here, we present a 3-D Brownian dynamics computational model of a cross-linked actin network including the dynamics of molecular motors and ACPs. The mechano-sensing properties of this active network are investigated by evaluating contraction and stress in response to different substrate stiffness. Results demonstrate two mechanisms that act to limit internal stress: (i) In stiff substrates, motors walk until they exert their maximum force, leading to a plateau stress that is independent of substrate stiffness, whereas (ii) in soft substrates, motors walk until they become blocked by other motors or ACPs, leading to submaximal stress levels. Therefore, this study provides new insights into the role of molecular motors in the contraction and rigidity sensing of cells.

## Introduction

Cells modulate their properties and activities in response to the surrounding environment, via morphological rearrangements driven by cytoskeletal contractility and reorganization. Various quantitative studies using gels with tuned elasticity have provided insights into the understanding of how cells respond to matrix stiffness [1], [2], [3], [4]. On soft substrates, cells generate low forces with randomly aligned actin filaments, leading to a weak response with wrinkles or strains of the substrates. By contrast, stiff substrates result in extensive cell spreading and enhance contractility with numerous stress fibers. Other experimental results collectively suggested that on stiff substrates, cells tend to deform intracellular structures rather than the substrate as seen in myosin/actin striations [5], [6], [7], [8].

Several mechanisms governing such mechano-sensing of cells have been proposed in experimental studies, and multiple mechanisms likely exist involving different intracellular structures. For example, a large number of mechano-sensing molecular motifs that vary conformation over a range of mechanical forces transduce mechanical signals into biochemical ones [9], [10], [11]. It has also been believed that actomyosin contractility contributes to cell mechano-sensing [12], [13], [14]. For example, non-muscle myosins were shown to be crucial for stem cells to sense matrix elasticity [15]. Local forces acting on both integrin-mediated [16], [17] and cadherin-mediated adhesions exhibit a similar relationship with stiffness [18], [19].

Different phenomenological laws have been proposed to explain the substrate-dependent mechano-sensing. For example, a simple “two-spring model” predicted that stiffer environments lead to stronger traction forces [20]. A “three-spring model” was proposed later to explain the stiffness-dependent orientation of stress fibers in adherent cells [21]. To elucidate interactions of molecular motors with adhesion complexes in the mechano-sensing process, a different theoretical model based on active matter theory was proposed [22]. It demonstrated that for short timescales (t≪100 s), mechano-chemical transduction from the motors plays a dominant role since the adhesion complexes are unlikely to have enough time to recruit associated proteins. Concurrently, numerous computational models have been developed to elucidate the mechanisms of mechano-sensing. For instance, they showed that actin networks can adjust to mechanical environments by modulating cross-links within the networks [23], and also suggested a mechanism for stiffness-sensing of cells adhered to a compliant surface mediated by actin filament alignment in the direction of force application [24].

Taken together, these recent experimental, theoretical, and computational efforts have led to new insights about the structural reorganization of the cytoskeleton as well as the effects of extracellular stiffness on cell behaviors. However, little is known about the roles of actomyosin contractility in mechano-sensing on timescales of hundreds of seconds, which are biologically relevant. In this work, using a Brownian dynamics computational model [25], [26], we investigate the large-scale contractile responses of an actomyosin network on timescales of hundreds of seconds, during which protein recruitment and responses from molecular motifs can occur, to elucidate one actomyosin-driven rigidity-sensing mechanism that functions under diverse conditions. Specifically, the effects of external elasticity on cytoskeletal contractility and network morphology are evaluated by systematically varying model parameters, e.g. the concentration and kinetics of motors. Our simulations successfully reproduce some of the large-scale mechano-sensing responses of cells such as active contractility and force generation, in good agreement with recent experimental observations [12], [27], [28]. Merely by modeling actin and myosin activity in the absence of proteins related to adhesion complexes, we predict both equilibrium and dynamic behaviors, indicating that actomyosin machinery can function as a stand-alone mechanism for the mechano-sensing of cells.

## Model

We use a previous agent-based model [26] based on Brownian Dynamics to simulate active cross-linked actin networks as systems that generate force as well as sense surrounding mechanical conditions. In this approach, we explicitly take into account actin filaments, ACPs, and molecular motors and their local interactions. To facilitate understanding of the results predicted in this study, we briefly present their main features.

### Formation of an Active Actin Network

**Figure 1:**
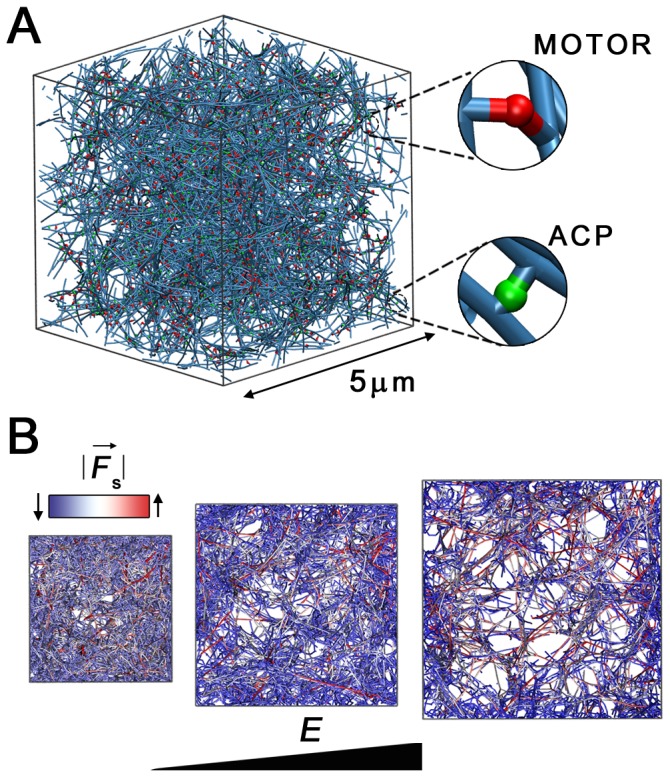
Illustration of the network morphology with different levels of substrate stiffness (*E*). The details of motors and ACPs are magnified. (**A**) The initial network is generated using a polymerization model [25], [26] and consists of actin filaments (cyan) cross-linked by ACPs (green) and molecular motors (red). (**B**) Cross-sections of the network at *t* = 200 s for three different values of *E* showing morphology and the magnitudes of extensional forces (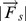). Soft substrates (lower *E*) lead to a condensed network, whereas stiff substrates (higher *E*) contract very little, resulting in a heterogeneous network with tensed filaments.

Active actin networks with motors are generated in a similar fashion to previous studies [26]. Monomers of actin (G-actins), passive ACPs, and motors are assembled into a network via reversible reactions in a 3-D cubical domain with periodic boundary conditions in all directions. Actin can exist in either monomeric or filamentous form while ACPs and motors can exist in three states: monomeric (free), inactive (partially bound), and active (bound to two filaments) states. Note that following the initial formation of the network, monomeric ACPs and motors are implicitly considered via their local concentration and second-order reaction equations. After concentrations of G-actin, ACPs, and motors reach a dynamic steady state, residual G-actins are deleted with actin assembly/disassembly deactivated for simplicity. A geometrically identical network is used in all simulations to isolate the effects of the stiffness of the surrounding medium and other parameters (Figure 1A). To vary the concentration of motors, they are removed from networks or added as monomers at the beginning. The average filament length (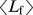) is ∼2 µm, actin concentration, *C*_A_, is 12 µM, density of ACPs, *R*_ACP_ ( = *C*_ACP_/*C*_A_), is 0.01, and the initial width of the cubical domain is 5.0 µm. Density of motors, *R*_M_ ( = *C*_M_/*C*_A_), is 0.02 unless specified.

### Mechanics of Actin Filaments, ACPs, and Motors

Actin filaments comprise cylindrical segments of length 140 nm (*r*_0,A_), and both the ACPs and motors are represented by two arms parallel to each other spanning between cross-linked actin filaments a distance of 70 nm (2×*r*_0,ACP_) and 140 nm (2×*r*_0,M_), respectively. Motions of the network components are governed by the Langevin equation:
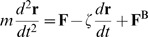
(1)where *m* is the mass of each element (actin, ACP, or motor), **r** is the element's location, *ζ* is the friction coefficient, *t* is time, **F** ^B^ is a thermal force satisfying the fluctuation-dissipation theorem, and **F** is a net deterministic force including extension, bending, and repulsive forces. Since inertia of all elements is negligible on the length and time scales of interest, positions of the elements are updated using the Euler integration scheme:
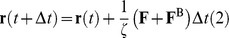
(2)where Δ*t* is a time step.

Extension and bending of the cylindrical segments constituting actin filaments, ACPs, and motors are computed using simple quadratic potentials, denoted by subscripts “s” and “b” respectively:
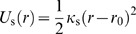
(3)

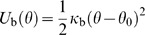
(4)where *r* is bond length, *κ*_s_ is extensional stiffness, *θ* is bending angle, *κ*_b_ is bending stiffness, and the subscript 0 denotes an equilibrium (zero-force) value. As in our previous studies [26], bending stiffnesses are introduced to restrict actin filament bending (*κ*_b,A_), keep the two arms of ACP (*κ*_b,ACP,1_) or motor (*κ*_b,M,1_) parallel, and maintain the right angle between the axis of a filament and the arm of ACP (*κ*_b,ACP,2_) or motor (*κ*_b,M,2_). Specific values of the geometrical and mechanical parameters are listed in Table S1. In addition, the repulsive force is responsible for volume-exclusion effects by which actin filaments cannot pass through each other, which is calculated by the following harmonic potential, *U*_r_, depending on the minimum distance, *r*_12_, between two cylindrical segments [26]:
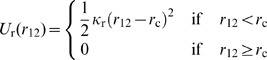
(5)where *κ*_r_ is the strength of repulsive effects, and *r*_c_ is the diameter of cylindrical segments. Then, the repulsive force is distributed to the two ends of the actin segment based on the relative location on the segment where *r*_12_ is measured.

### Dynamic Behaviors of ACPs and Motors

We assume that each motor in our simulation corresponds to a single myosin minifilament consisting of multiple myosin II molecules. As described in our previous studies [29], motors in the active state walk along actin filaments toward a barbed end at a rate, *k*_w_, depending on the extensional force acting on the arm, 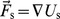:
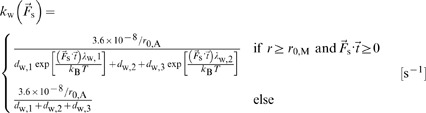
(6)where *d*_w_'s and *λ*_w_'s are time constants and mechanical sensitivities for walking of motors (Table S1), respectively, and 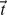 is a unit vector locally tangent to an actin segment in the direction of a pointed end. Although motors in this study mimic a myosin minifilament consisting of numerous myosin II molecules, Eq. 6 and the values of *d*_w_'s and *λ*_w_'s are adopted from a single-molecule experiment examining myosin V under 1 mM ATP. Our intention was to model generalized motor activity, and we chose myosin V because it has been extensively characterized. Nevertheless, the load-dependent walking rate of the minifilament is still qualitatively similar to that of myosin V, justifying the use of Eq. 6 for roughly mimicking myosin minifilament behavior. As seen in Eq. 6, only tension (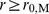) directed to a pointed end (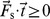) affects *k*_w_, resulting in a stall force, ∼4 pN, beyond which motors cease walking.

In addition, as in [30], ACPs and motors are able to unbind in a force-dependent manner following Bell's equation:
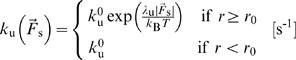
(7)where 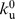 is the zero-force unbinding rate coefficient for ACPs (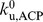) or motors (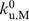), and 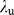 is the mechanical sensitivity for unbinding of ACPs (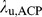) or motors (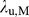) (Table S1). Note that although unbinding of motors is also one of the phases of walking, these two events are considered separable for systematic analysis. If the arm of motors reaches the barbed end of a filament by walking, it remains there until it unbinds.

### Boundary Conditions of the 3-D Computational Domain

After obtaining the network, actin filaments crossing the domain boundaries are severed and permanently clamped with periodic boundary conditions deactivated in all directions. During the measurement of strain and stress, the boundaries also act as sticky surfaces to take the binding between actin filaments and membrane into account; if either end of an actin filament is located within 30 nm of a boundary, the end is irreversibly clamped.

Normal stress (*σ*) on each boundary is the sum of normal forces exerted by actin filaments clamped on the boundary, divided by area. *σ* is used to compute movement of the boundaries in simulation; we assume that the domain is surrounded by an elastic medium with identical Young's modulus, *E*, on all boundaries. Each boundary (assumed planar) experiencing *σ* is displaced a distance corresponding to a strain *σ*/*E*.

Simulations begin with zero stress on all boundaries and proceed over time for 200 s. At this point, the network reaches a steady state stress in most cases and we define this to be the plateau stress. However, in a few cases, stress continues to slowly rise even after 200 s.

### Measurement of network stiffness

In order to measure the stiffness of networks, we applied differential sinusoidal normal displacement of amplitude 280 nm to the networks and calculated the responding stress. For the purpose of this calculation, all the motor and actin cross-linking dynamics was deactivated to probe the instantaneous network stiffness, avoiding any progressive time-dependent changes in the network. Under these conditions, the networks exhibit a predominantly elastic response as indicated by the small phase delay between the applied strain and the responding stress (Figure S1). We then calculated network stiffness by dividing the amplitude of stress by that of strain.

## Results

Here, we investigate the role of molecular motors as rigidity sensors and predict the contractile (normal) stress and strain of actomyosin networks tethered to 3-D cubical domains. We examined these as a function of the various kinetic parameters and concentrations of motors as well as different elasticity of the surrounding medium.

### Network morphology and stress evolution depend on substrate stiffness

**Figure 2:**
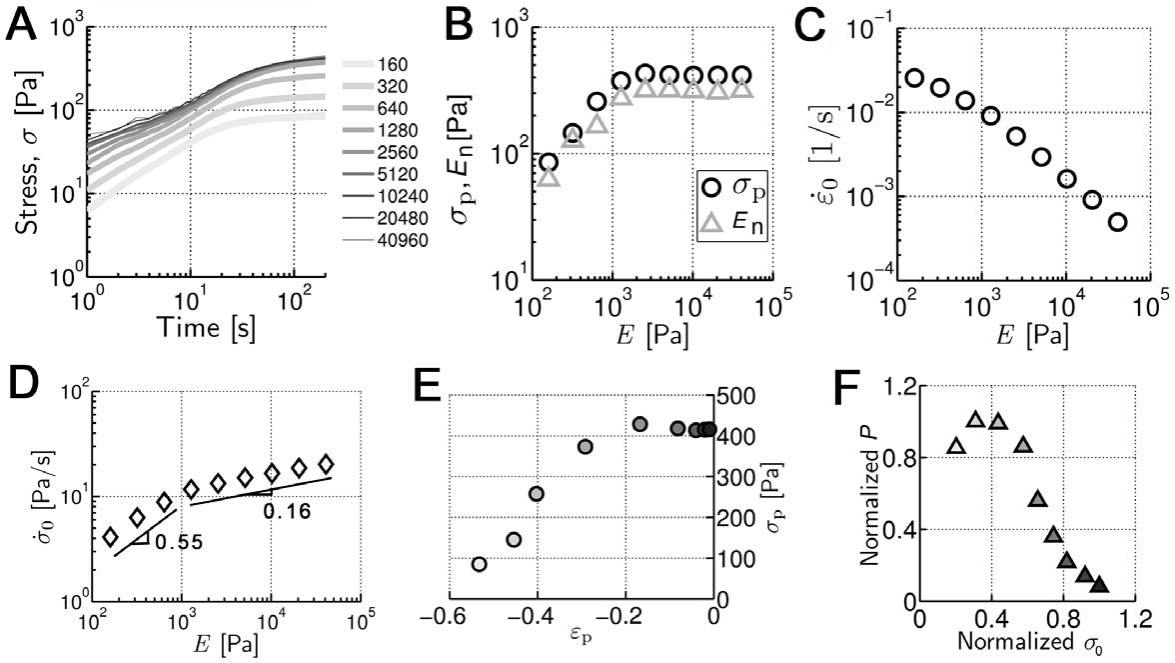
Effects of *E* on stress ( 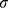
 **) and strain (** 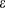 **) behaviors.** (**A**) Time evolution of 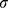 at different *E*. Numbers in the legend indicate the values for *E*. 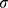 increases rapidly at first but reaches a nearly constant plateau value (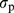) at ∼200 s regardless of *E*. (**B**) 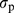 (circles) and network stiffness (*E*_n_, triangles) as functions of *E*. 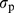 monotonically increases for *E*<3 kPa but saturates for *E*>3 kPa. The network stiffness shows the same tendency as 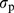 for all *E*. (**C**) Contraction speed (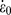) as function of *E*. The network contracts rapidly with low *E* but more slowly as *E* increases. (**D**) Initial rate of stress increase (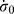) with different *E*. 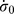 increases following 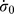 **∼** *E* ^0.55^ for *E*<1 kPa and 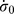 **∼** *E* ^0.16^ for *E*>1 kPa. (**E**) 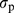 and corresponding strain (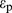) at various *E*. Higher 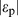 corresponds to lower 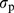. For high *E*, 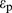
asymptotically approaches 0. (**F**) A relation between normalized power (*P*) and initial stress (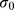). *P* becomes maximal at *E*∼0.6 kPa, generating 40% of the maximum 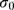 and intermediate 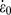 of ∼0.015 s^−1^. Each color within the symbols in **E** and **F** indicates the value of *E* in **A** with the same line color.

The initial network (Figure 1A) starts from a zero stress condition. Due to motor activity, and depending on substrate stiffness (*E*), the network shrinks to different extents at different rates. Lower *E* leads to shrunk and concentrated networks with highly bent actin filaments (Figure 1B). On the other hand, higher *E* prevents the domain contraction, forming heterogeneous networks with highly stretched filaments (Figure 1B). These differences in network morphology have been reported in experiments where they found, for different cell types, that F-actin networks tend to be denser and less organized on more compliant substrates [31], [32]. Stress (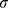) in all cases rapidly increases at the beginning although the rate of increase gradually falls, rising at a much slower rate by ∼200 s in most cases (Figure 2A). Recognizing that stress continues to rise after this time, but constrained by computational resources from extending the calculations further, we use the value of stress at 200 s as a reference, and denote it as the “plateau stress”, 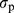. For *E*<3 kPa, 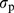 is proportional to *E* but becomes relatively constant for *E*≥3 kPa, which corresponds well to literature [3], [12], [16], [27] (Figure 2B). The maximum of 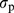 is ∼420 Pa. The initial slope of stress, 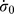, measured at *t*<10 s increases swiftly for *E*<3 kPa and slower for *E*>3 kPa (Figure 2D). The initial strain rate, 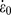 ( = 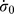/*E*), decreases with greater *E* (Figure 2C); since contraction is associated with energy expenditure to overcome the internal friction and the rupture of cross-links, cells contracting against softer substrates will experience larger energy dissipation, leading to the slower rise in stress. The “plateau strain”, 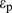 (strain at plateau stress), decreases with greater *E* falling below 0.05 for *E*>10 kPa (Figure 2E). 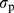 and 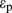 with various *E* show a first zone where 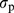 rapidly changes, followed by a period of slower increase, which agrees well with [33]. We also measured mechanical power, *P* = 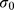 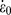 *V* where 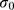 is initial stress corresponding to 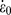, and *V* is the instantaneous volume of the domain. *P* exhibits a bimodal dependence on 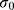, having a peak at *E*∼0.6 kPa (Figure 2F). At this peak, the network exerts 40% of the maximum 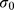 with intermediate 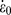 (∼0.015 s^−1^), compared to cases with other *E*.

### Network stiffness tracks the generated stress

It has been recently found that cell stiffness tracks substrate stiffness over a range of stiffnesses before reaching a constant value [34]. We measured the steady-state stiffness of networks at each *E*. Network stiffness (*E*_n_) was found to be proportional to (and nearly equal to) 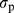 over the entire range of *E*, but proportional to *E* only up to a value of *E*∼3 kPa (Figure 2B). This tendency is consistent with the direct proportionality between prestress and *G*′ (or *K*′) of passive actin networks observed in experiments [35].

### Effects of motor concentration

**Figure 3:**
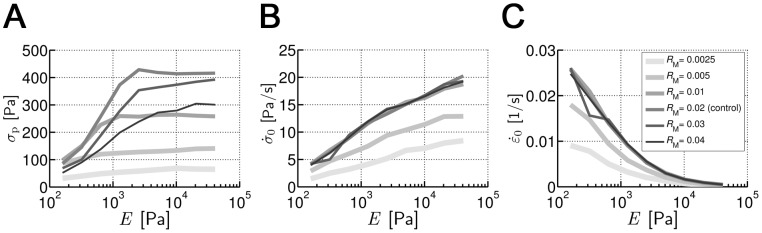
Influences of motor concentration (*R*_M_). (**A**) 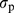
(*E*) with various *R*_M_. With low *R*_M_, the tendency of an increase followed by a plateau is less clear. 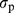 is maximal for all *E* at either *R*_M_ = 0.01 or 0.02. (**B**) 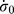
(*E*) and (**C**) 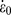
(*E*). Both 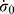 and 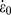 increase with greater *E* and are higher for greater *R*_M_ until saturation at *R*_M_≥0.02. The legend in **C** is also applicable to **A** and **B**.

Motor density is varied by adjusting the initial concentration of motors in the network, *R*_M_. In all cases, 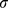 increases with *E* and then exhibits a much slower rate of increase at high *E* (Figure 3A), but compared to the control case (*R*_M_ = 0.02), the tendency is less clear in the other cases, especially for low *R*_M_ where the dependence between 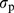 and *E* weakens. For low *R*_M_ (<0.02), 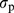 tends to be higher with greater *R*_M_, in good agreement with literature [12], [36], [37]. With a maximum at *R*_M_ = 0.02 for most *E*, 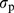 drops for higher *R*_M_. High contractile activity of the network enhances the rate of stress generation. Thus, 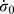 and 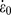 increase for all values of *E* until *R*_M_ reaches the optimal level explained above (Figure 3B and C).

### Effects of unbinding and walking behaviors of motors

Motor unbinding is explored by varying the zero-force unbinding rate (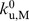) and the processivity (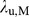). On the other hand, motor walking is studied by varying the sensitivity (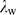) which is equivalent to variation of the stall force.

Although higher 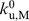 results in more frequent unbinding, it does not necessarily lead to lower 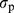 since unbinding can also help stalled motors due to blocking effects to bind to other binding sites so that they can keep walking. However, too frequent unbinding prevents motors from remaining attached to filaments for enough time to generate large stress. The early phase of 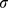 evolution is practically unaffected by changes in 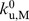 while the later phase is strongly influenced; 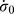, 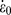 and *P* are relatively conserved, whereas 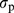 tends to decrease with higher 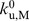 (Figure S2A–D). These are likely to account for the complicated effects of 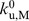.

**Figure 4:**
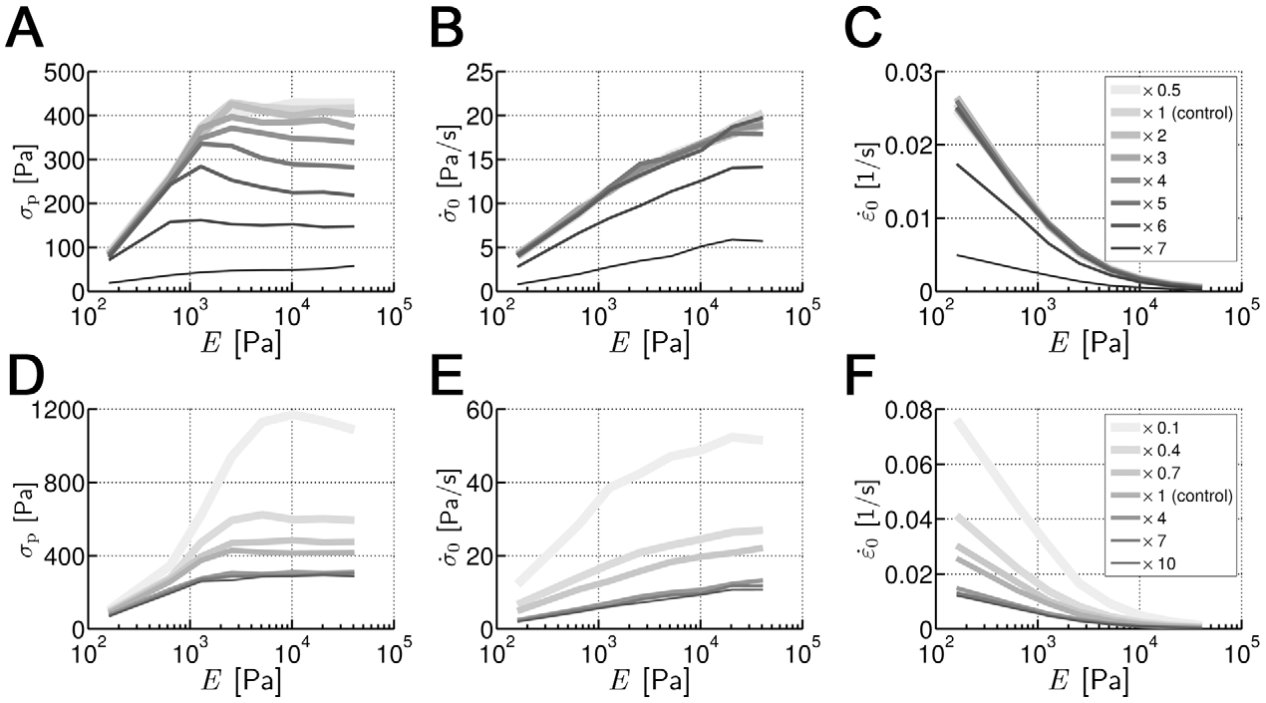
Effects of mechanical sensitivity of motor unbinding and motor walking. (**A–C**) Influence of the mechanical sensitivity of motor unbinding (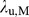 = *n*×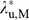) and (**D–F**) motor walking (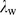 = *n*×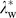) on (**A, D**) 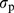
(*E*), (**B, E**) 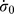
(*E*), and (**C, F**) 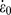
(*E*). “*” denotes reference values (Table S1), and numbers in the legends indicate *n*. Note that **A** and **B** share a legend with **C**, and **D** and **E** share a legend with **F**. 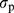 tends to be higher with lower 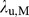 and 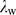 which correspond to more processive and stronger motors, respectively.

By contrast, the effects of 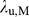 and 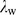 are much clearer; 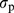, 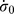, and 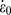 tend to be all higher for lower 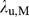 (Figure 4A–C) and 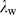 (Figure 4D–F). Note that 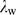 refers to both 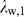 and 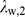, and their values are varied simultaneously. In response to variations of 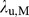, the typical tendency of 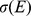 is conserved in most cases except that with 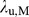 = 7×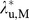 where the dependence on *E* is noticeable only at low *E* since motors can bear very small forces (Figure 4A). Therefore, 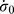 and 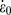 deviate from the control case only for high values of 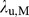 (Figure 4B and C). Interestingly, *P*(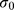) and 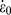
(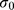) after normalization are collapsed into a unique curve (Figure S3A and C). Regarding motor walking, 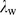 determines the stall force at which the walking rate of motor approaches nearly zero (Eq. 6). Lower values of 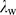 lead to higher stall forces, so the motors can overcome the applied forces and thus walk longer distances. Multiplying 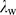 by 0.1 increases 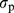 nearly three times compared to the control case (Figure 4D). Besides, due to higher 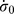, the domain shrinks more notably (Figure 4E and F). Again, *P*(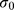) and 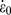
(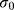) collapse well into a single curve after normalization (Figure S3B and D).

## Discussion

Cells are capable of adapting their properties through a variety of mechanisms. In this study, we investigated the role of molecular motors as rigidity sensors and also propose how these motors can induce such precise mechano-sensing. Despite the oversimplifications and assumptions made in the described system which only includes the dynamics of motors and ACPs, the simulated actin network exhibits macroscopic contractile behaviors remarkably similar to several experiments [12], [27], [28], further indicating that microscopic properties of individual constituents govern the network responses, and that motors play a central role in mechano-sensing. However, this does not negate the significance of other factors such as actin dynamics and structures, biochemical signaling, and adhesions dynamics in the cell's response and adaptation to mechanical cues. Rather, we demonstrate here that actomyosin machinery can be one of several possible mechanisms for cell rigidity-sensing phenomena. Nevertheless, we explored a wide range of parametric spaces in order to study how different parameters of the model influence cell adaptation, finding that those parameters affecting the kinetics of motors are the most critical for cellular adaptation to substrate stiffness.

**Figure 5:**
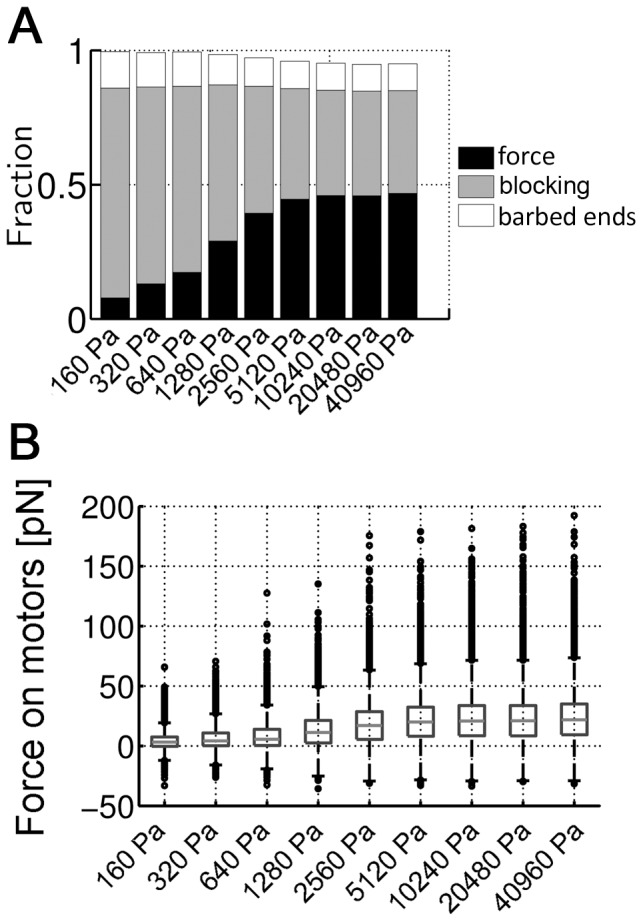
Mechanisms limiting network contraction. (**A**) Fraction of motors stalled at steady state (*t* = 200 s) due to three different reasons as a function of *E*: high applied forces (black), lack of binding sites (blocking, gray), or arrival at barbed ends of filaments (white). The blocking effect is a major cause of motor stalling at all *E*. Percentage of motors stalled by applied loads increases with *E* for *E*<3 kPa and becomes independent of *E* for *E*>3 kPa. Since the number of barbed ends in the domain is constant, the corresponding fraction is largely independent of *E*. (**B**) Statistical distribution of forces acting on motors at *t* = 200 s. The median slightly increases with *E*, whereas the upper quartile clearly increases at higher *E*.

We probed diverse contractile large-scale characteristics by evaluating network morphology, plateau stress (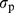), the initial increasing rate of stress (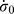), the initial rate of strain (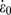), and mechanical power (*P*) over a wide range of parameters – the stiffness of the surrounding environment (*E*), the concentration (*R*_M_) and the dynamics of motors (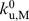, 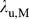, and 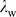). It was observed that softer substrates lead to shrunken and dense networks, whereas stiffer ones result in heterogeneous networks with minimal domain contraction (Figure 1B). Overall, the qualitative pattern of stress evolution is quite consistent with multiple recent experimental works [12], [14], [27]; 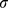 increases with time rapidly at first but reaches 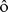 in most cases before 200 s (Figure 2A). In addition, we found that 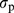 is roughly proportional to *E* for *E*<3 kPa and becomes relatively constant for *E*>3 kPa (Figure 2B). The existence of a transition to a slower rate of stress increase can be explained by the mechanisms that cause the motors to slow or stall: (i) all of the next binding sites in a barbed-end direction are already occupied (blocking), (ii) reaching the motor stall force, or (iii) reaching the barbed end of an actin filament. Figure 5A demonstrates that only a small fraction of motors reach the barbed end of a filament for all *E*, so this would have little direct influence on 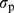. Blocking, on the other hand, is observed over the entire range of *E*, but is especially prevalent at lower *E*. This is due to the greater distance that motors need to walk before reaching their maximum force, combined with the tendency for all constituents (filaments, motors, and ACPs) to increase in density under large negative strains. For stiffer substrates, material strains are smaller and motors walk shorter distances before attaining the stall force. For *E*>3 kPa, 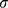 can reach 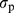 determined by the stall force that motors can exert maximally while at lower *E*, 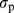 is limited by the blocking effect which progressively decreases as *E* increases (Figure 5B). This transition from blocking at low *E* to limitation due to motor stall force at high *E* constitutes a mechanism by which cells can sense substrate stiffness. Forces transmitted along the cytoskeleton and across adhesion complexes will vary according to the generated stress, leading to varying degrees of conformational change in these stress-bearing proteins. Since conformation determines biochemical activity, factors such as exposing cryptic binding sites, changes in binding affinity, or phosphorylation, for example, will modulate signaling activity and therefore, cell function.

This mechanism is further confirmed by effects of *C*_A_ on 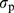 (Figure S4). At *E* = 2560 Pa, we varied *C*_A_ but maintained *C*_M_ at 0.24 µM, meaning that *R*_M_ decreases with higher *C*_A_. Note that *C*_M_ = 0.24 µM is equivalent to *R*_M_ = 0.02 in the control case with *C*_A_ = 12 µM. 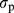 is proportional to *C*_A_ even with the same number of motors (Figure S4A). More actins can provide the motors with greater space to walk, leading to fewer stalling events by blocking but more frequent stalling by applied forces (Figure S4B). In addition, the effects of average filament length (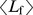) demonstrate the mechanism. We found that networks attain stress roughly proportional to the cube of 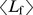 (Figure S5A). With shorter filaments, more motors reach the barbed ends while with longer filaments, motors are more likely to stall by attaining their maximum level of force generation (Figure S5B). Interestingly, the percentage of blocking events remains relatively constant, independent of 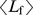. The increase in the number of motors exerting maximum stall forces results in higher 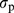, consistent with our mechanism above.

Several recent studies suggested that the proportionality between 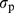 and *E* could be correlated with 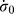 [12], [27], [28], which is consistent with our observation that 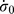 increases swiftly for *E*<3 kPa but slows down for *E*>3 kPa (Figure 2D). From a biological point of view, cells are polarized and migrate in the direction of higher stiffness inducing the faster increase of traction force [3], [38], [39], [40], [41]. The rise of 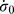 with increasing *E* is in the same order of magnitude of the experimental findings [12], [27], [28]. Although the time required to reach 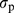 is somewhat different between ∼200 s in our simulations and ∼600 s in experimental studies, it is common that the time needed to reach 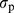 is relatively independent of *E* in both, as found in [12] (Figure 2A). The inverse proportionality between 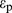 and *E* (Figure 2E), consistent with experiments [33], supports that cells on stiff substrates tend to rearrange intracellular structures rather than deforming the substrate, as in [5], [6], [7], [8]. Numerical results of 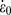 depending on *E* (Figure 2C) and of *P*(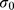) (Figure 2F), show that the emergent behavior of the network follows Hill's equation for muscle contraction [42]. We fit the 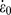
(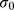) curve (Figure S6) with the relation 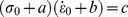 where *a* = 10.0 Pa, *b* = 0.0103 s^−1^, and *c* = 1.0023 Pa/s. In experiments using various types of muscle cells [43] and myoblasts cells [12], introducing a shape factor 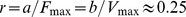 normalized the data. For our control case, we obtained 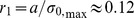 and 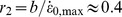. We found that the values of these factors are regulated by parameters, such as 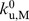, 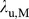, and 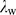, although normalizing the data leads to similar curves (Figure S3A–D).

We provided further insights regarding the effects of motor concentration (*R*_M_). It was observed 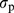 attains a maximum at *R*_M_ = 0.02 (Figure 3A). This could be attributed to the limited binding sites for motors on actin filaments in our simulations. However, note that *R*_M_ in our model corresponds to the concentration of multimerized myosin II structures in cells rather than that of individual molecules. Therefore, *R*_M_ = 0.02 is actually a very high density of large motor structures, and thus cells are likely to have such an optimal *R* due to blocking effects between the large aggregates of myosins. A refined model including multiple myosin heads per motor and multiple binding sites per actin segment would help to clarify this issue.

Considering the variable extent of multimerization of myosin II molecules into a minifilament or thick filament, we evaluated the effects of the zero-force rate of motor unbinding (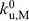) (Figure S2) and the mechanical sensitivity for unbinding (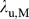) and walking (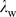) on network contraction. Motors with high 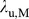 more readily unbind, and those with high 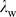 are more likely to be stalled at small forces. 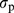, 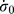, and 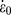 are higher for lower 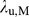 (Figure 4A–C) or for lower 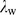 (Figure 4D–F) although the overall trend of the curves is conserved well in both cases. By contrast, with high 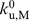, 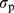 is substantially reduced while 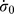 and 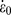 are not affected (Figure S2A–C).

## Conclusion

We elucidated one mechanism by which cells can modulate their properties and respond to the surrounding environment via cytoskeleton contractility, using an agent-based computational model. Although the model is based on molecular-level processes, macroscopic behaviors of the active cross-linked actin networks agree well with the response of cells probed in experimental quantitative studies [1], [2], [3], [4]. We found that the biphasic relation between substrate stiffness and the level of generated forces [5], [6], [7], [8] is attributable to a transition from stalling due to steric hindrance or “blocking” in soft substrates to that due to stall forces in stiff substrates. In addition, we showed that in response to increases in substrate stiffness, the contraction rate of cells increases while the corresponding contraction velocity decreases, also consistent with experiments [28]. All of these suggest that actomyosin contractility is one plausible stand-alone mechanism capable of contributing directly to cell mechano-sensing [12], consistent with various experimental findings that myosins are crucial for cells to sense surrounding matrix elasticity [14], [15], and that cell responses to rigidity of the external matrix reflect adaptation of the actomyosin machinery to load following Hill's relation [12].

## Supporting Information

Figure S1 **Measurement of network stiffness.** (A) Sinusoidal normal strain applied to networks to measure the steady-state stiffness of networks (*E*_n_), corresponding to an amplitude of 280 nm. (B) Stress in response to the applied strain. These show examples of stress and strain for a control case with *E* = 40960 Pa.
(TIF)

Figure S2
**Influences of zero-force unbinding rate of motors.** Effects of 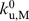 ( = *n*×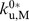) on (**A**) 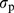
(*E*), (**B**) 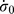
(*E*), (**C**) 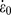
(*E*), and (**D**) *P*(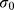). Numbers in the legends represent *n*, and **A**, **B** and **C** share the same legend. The early phase of stress evolution is virtually unaffected by changes in 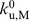 while the later phase is strongly influenced. This means that 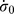, 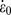 and *P* are relatively conserved (**B–D**), whereas 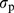 tends to decrease with higher 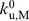 (**A**), demonstrating that motor unbinding plays a role only in determining the level of stress that can be attained under steady-state conditions once stress has developed. There also appears to be an optimal stiffness (at least for high 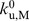) at which plateau stress reaches a maximum.(TIF)

Figure S3
**Influences of mechanical sensitivity of motor unbinding and walking.** (**A, C**) motor unbinding (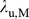 = *n*×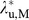) and (**B, D**) motor walking (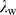 = *n*×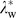). Numbers in the legends indicate *n*. **A** shares a legend with **C** (unbinding); and **B** shares a legend with **D** (walking). (**A, B**) and (**C, D**) show normalized *P* and 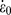 vs normalized 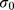, respectively. Regardless of *n*, the curves collapse well after normalization. *P* exhibits a biphasic behavior with a peak at ∼40% of 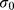. On the other hand, 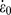 decreases with increasing 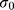, approaching zero for higher loads.(TIF)

Figure S4
**Effects of actin concentration (** ***C*** **_A_).** (**A**) 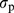 monotonically increases with *C*_A_. In these simulations, *R*_ACP_ is constant at 0.01, but *R*_M_ decreases with higher *C*_A_ since *C*_M_ is fixed at 0.24 µM, corresponding to the constant number of motors. (**B**) Fraction of motors stalled due to: (i) high applied forces (black), (ii) blocking (gray), or (iii) arrival at barbed ends of filaments (white) at steady state as a function of *C*_A_. At low *C*_A_, ∼50% of motors are not stalled since many of them lie in the inactive state due to lack of network percolation. As *C*_A_ increases, motors are more likely to be stalled due to high forces as opposed to blocking.(TIF)

Figure S5
**Effects of average actin filament length (** 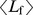 **).** (**A**) 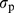 increases dramatically as 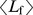 is increased. (**B**) Fraction of motors stalled due to: (i) high applied forces (black), (ii) blocking (gray), or (iii) arrival at barbed ends of filaments (white) at steady state as a function of 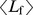. As 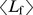 increases, more motors are stalled due to attaining their maximum force while fewer motors are stalled due to arrival at barbed ends. The number of motors stalled due to blocking remains nearly constant regardless of 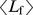.(TIF)

Figure S6
**Comparison of** 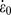 **(** 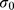 **) for the control case to Hill's equation.** The network shrinks faster for softer substrates, developing less stress while slower shrinkage leads to higher stress. Values for the constants *a*, *b*, and *c* in Hill's equation 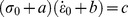 are 10.0 Pa, 0.0103 s^−1^, and 1.0023 Pa/s respectively. Note that 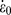 and 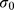 were measured at *t* = 10 s.(TIF)

Table S1 **List of model parameters.** Numbers in parentheses are corresponding dimensionless values as defined in the text. “*” on symbols indicates reference values of parameters studied in the sensitivity analysis. Values marked by “§” are adopted from given literature with adjustment based on assumption that motors in this study consist of many myosin II molecules. Note that to increase length (*N*_c_) and time scales (Δ*t*), *κ*_s,A_ used in this study is 4 times smaller than that in our previous works, but it was confirmed that the results are virtually unaffected by the 4-fold decrease.
(DOC)
