# Supplementary figures and images for: Dynamic Mechanisms of Cell Rigidity Sensing: Insights from a Computational Model of Actomyosin Networks
Source: PLoS One. 2012 Nov 5;7(11):e49174. doi: 10.1371/journal.pone.0049174 (PMC3489786; doi:10.1371/journal.pone.0049174)

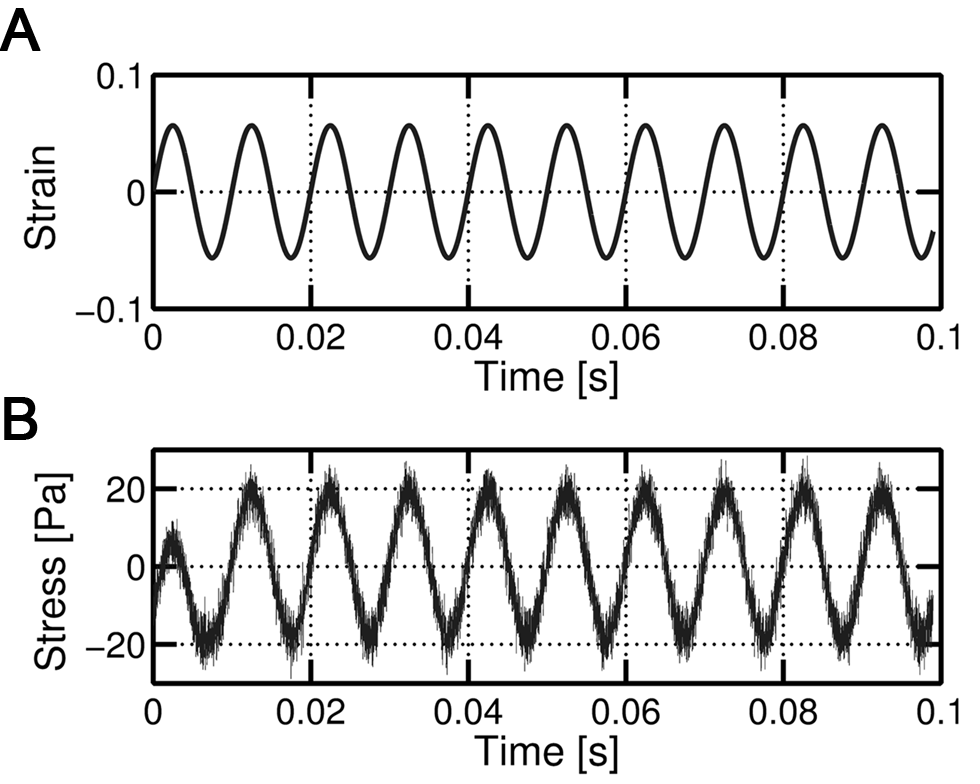

Supplement: Figure S1 — Measurement of network stiffness. (A) Sinusoidal normal strain applied to networks to measure the steady-state stiffness of networks (E n), corresponding to an amplitude of 280 nm. (B) Stress in response to the applied strain. These show examples of stress and strain for a control case with E = 40960 Pa. (TIF) [file pone.0049174.s001.tif]

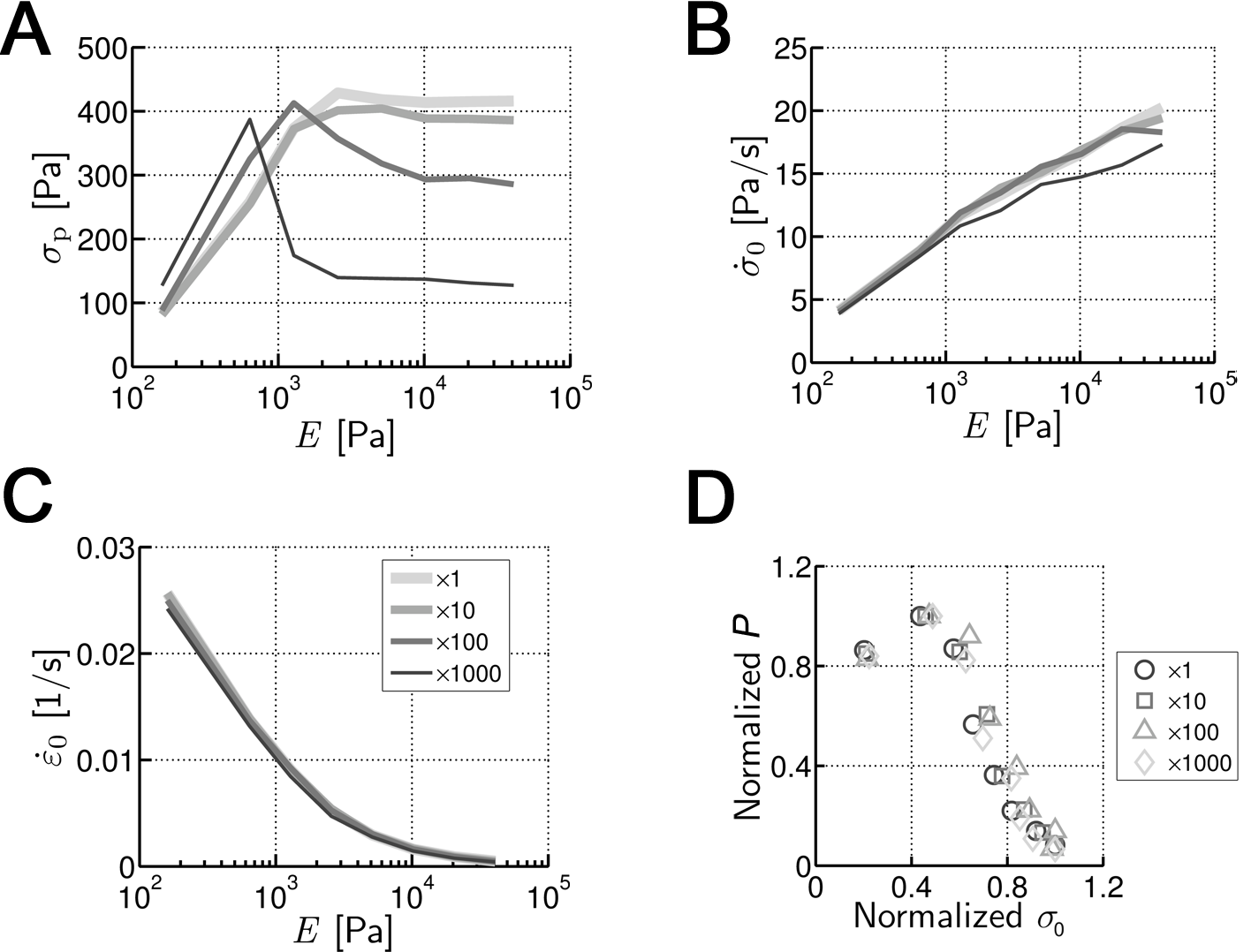

Supplement: Figure S2 — Influences of zero-force unbinding rate of motors. Effects of ( = n×) on (A) (E), (B) (E), (C) (E), and (D) P(). Numbers in the legends represent n, and A, B and C share the same legend. The early phase of stress evolution is virtually unaffected by changes in while the later phase is strongly influenced. This means that , and P are relatively conserved (B–D), whereas tends to decrease with higher (A), demonstrating that motor unbinding plays a role only in determining the level of stress that can be attained under steady-state conditions once stress has developed. There also appears to be an optimal stiffness (at least for high ) at which plateau stress reaches a maximum. (TIF) [file pone.0049174.s002.tif]

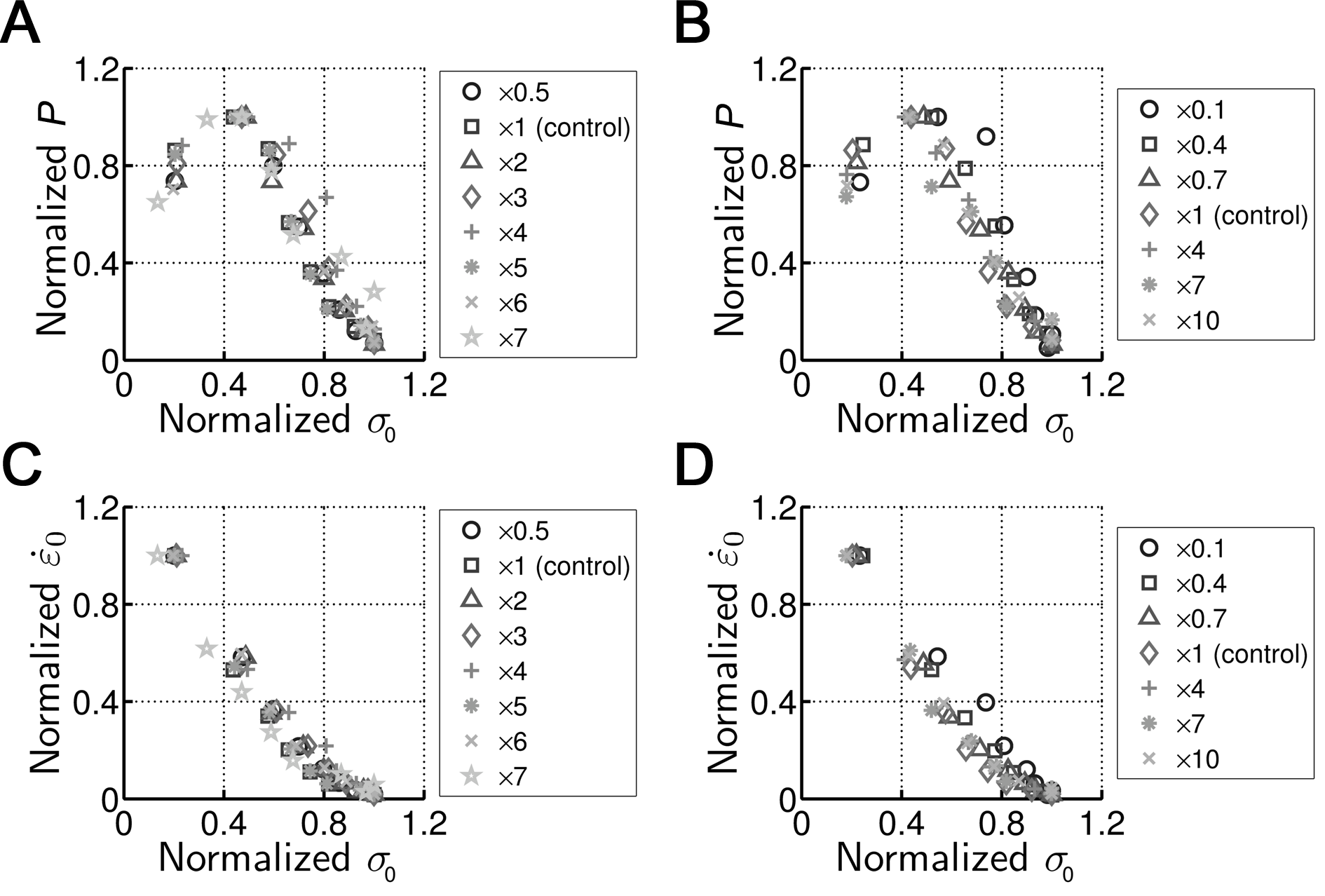

Supplement: Figure S3 — Influences of mechanical sensitivity of motor unbinding and walking. (A, C) motor unbinding ( = n×) and (B, D) motor walking ( = n×). Numbers in the legends indicate n. A shares a legend with C (unbinding); and B shares a legend with D (walking). (A, B) and (C, D) show normalized P and vs normalized , respectively. Regardless of n, the curves collapse well after normalization. P exhibits a biphasic behavior with a peak at ∼40% of . On the other hand, decreases with increasing , approaching zero for higher loads. (TIF) [file pone.0049174.s003.tif]

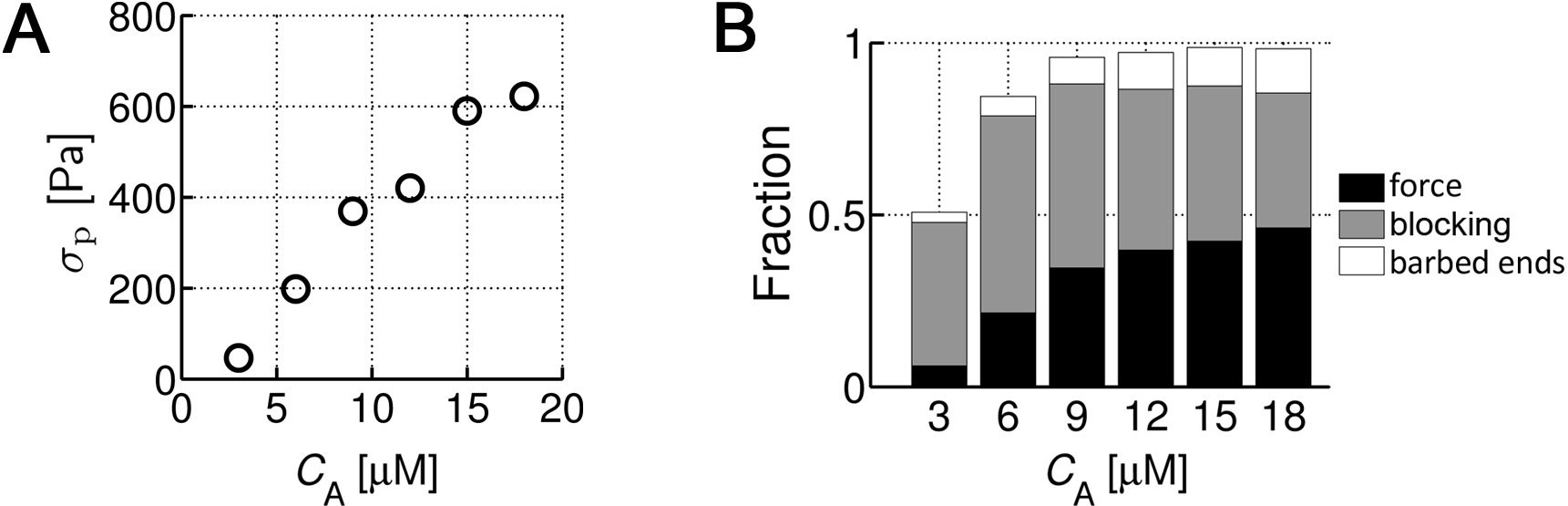

Supplement: Figure S4 — Effects of actin concentration ( C A). (A) monotonically increases with C A. In these simulations, R ACP is constant at 0.01, but R M decreases with higher C A since C M is fixed at 0.24 µM, corresponding to the constant number of motors. (B) Fraction of motors stalled due to: (i) high applied forces (black), (ii) blocking (gray), or (iii) arrival at barbed ends of filaments (white) at steady state as a function of C A. At low C A, ∼50% of motors are not stalled since many of them lie in the inactive state due to lack of network percolation. As C A increases, motors are more likely to be stalled due to high forces as opposed to blocking. (TIF) [file pone.0049174.s004.tif]

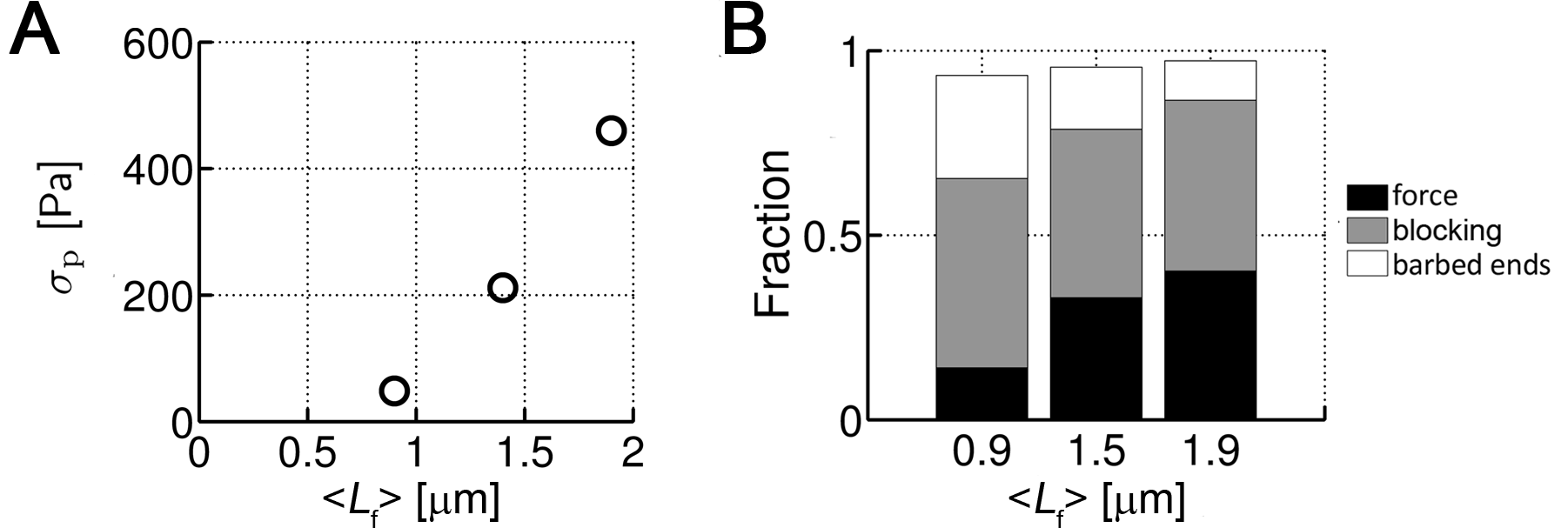

Supplement: Figure S5 — Effects of average actin filament length ( ). (A) increases dramatically as is increased. (B) Fraction of motors stalled due to: (i) high applied forces (black), (ii) blocking (gray), or (iii) arrival at barbed ends of filaments (white) at steady state as a function of . As increases, more motors are stalled due to attaining their maximum force while fewer motors are stalled due to arrival at barbed ends. The number of motors stalled due to blocking remains nearly constant regardless of . (TIF) [file pone.0049174.s005.tif]

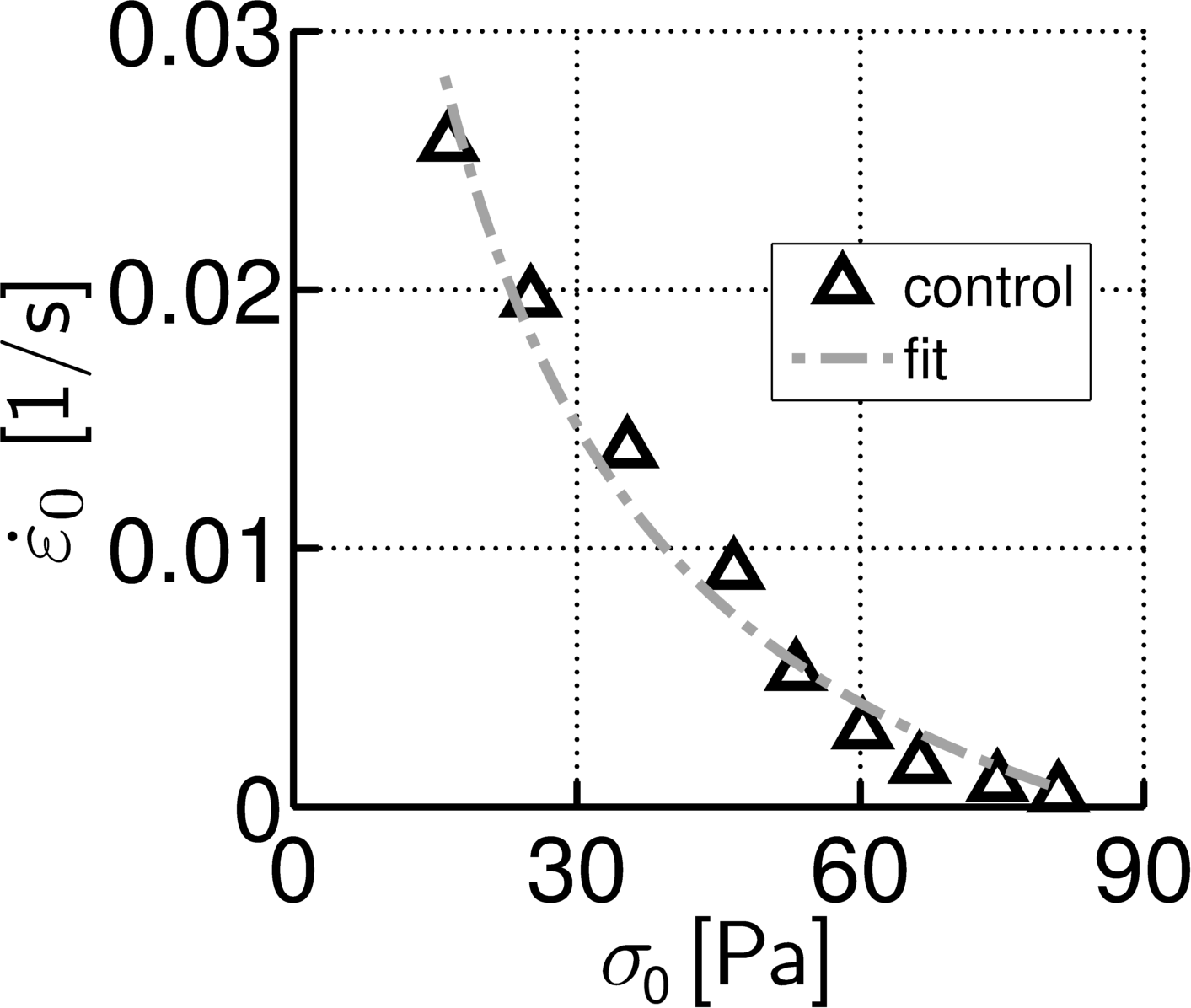

Supplement: Figure S6 — Comparison of ( ) for the control case to Hill's equation. The network shrinks faster for softer substrates, developing less stress while slower shrinkage leads to higher stress. Values for the constants a, b, and c in Hill's equation are 10.0 Pa, 0.0103 s−1, and 1.0023 Pa/s respectively. Note that and were measured at t = 10 s. (TIF) [file pone.0049174.s006.tif]
