# Supplementary material for: Dynamic Mechanisms of Cell Rigidity Sensing: Insights from a Computational Model of Actomyosin Networks
Source: PLoS One. 2012 Nov 5;7(11):e49174. doi: 10.1371/journal.pone.0049174 (PMC3489786; doi:10.1371/journal.pone.0049174)
Supplement: Table S1 — List of model parameters. Numbers in parentheses are corresponding dimensionless values as defined in the text. “*” on symbols indicates reference values of parameters studied in the sensitivity analysis. Values marked by “§” are adopted from given literature with adjustment based on assumption that motors in this study consist of many myosin II molecules. Note that to increase length (N c) and time scales (Δt), κ s,A used in this study is 4 times smaller than that in our previous works, but it was confirmed that the results are virtually unaffected by the 4-fold decrease. (DOC) [file pone.0049174.s007.doc]

| Variable | Symbol | Value |  |
| --- | --- | --- | --- |
| Diameter of cylindrical actin segments | *r*_c_ | 7.0×10^-9^ [m] (0.05) | |
| Length of cylindrical actin segments | *r*_0,A_ | 1.4×10^-7^ [m] (1.0) | |
| Time step | Δ*t* | 2.3×10^-8^ [s] (1.0×10^-5^ ) | |
| Strength of repulsive force | *κ*_r_ | 4.2×10^-4^ [N/m] (2,000) | |
| Extensional stiffness of actin | *κ*_s,A_ | 4.2×10^-3^ [N/m] (20,000) | |
| Bending stiffness of actin | *κ*_b,A_ | 2.64×10^-19^ [N m] (63.75) | |
| Number of actins that each segment represents | *N*_c_ | 20 | |
| Length of a single arm of ACP | *r*_0,ACP_ | 3.5×10^-8^ [m] (0.25) | |
| Extensional stiffness of ACP | *κ*_s,ACP_ | 4.3×10^-4^ [N/m] (2,000) | |
| Bending stiffness 1 of ACP | *κ*_b,ACP1_ | 1.04×10^-18^ [N/m] (250) | |
| Bending stiffness 2 of ACP | *κ*_b,ACP2_ | 4.142×10^-18^ [N m] (1,000) | |
| Length of a single arm of motor | *r*_0,M_ | 7.0×10^-8^ [m] (0.5) | |
| Extensional stiffness of motor | *κ*_s,M_ | 4.23×10^-4^ [N/m] ] (2,000) | |
| Bending stiffness 1 of motor | *κ*_b,M1_ | 1.04×10^-18^ [N/m] (250) | |
| Bending stiffness 2 of motor | *κ*_b,M2_ | 4.142×10^-20^ [N m] (10) | |
| Substrate stiffness | *E* | 0.16 - 40.96 [kPa] | |
| Concentration of actin | *C*_A_ | 1.2×10^-5^ [M] | |
| Ratio of *C*_ACP_ to *C*_A_ | *R*_ACP_ | 0.01 | |
| Ratio of *C*_M_ to *C*_A_ | *R*_M_ | 0.02 (unless specified) | |
| Zero-force unbinding rate coefficient of ACP | $k_{\text{u,ACP}}^{0}$ | 0.115 [s^-1^] | |
| Sensitivity of ACP unbinding | $\lambda_{\text{u,ACP}}$ | 1.04×10^-10^ [m] | |
| Zero-force unbinding rate coefficient of motor | $k_{\text{u,M}}^{0*}$ | 2×10^-5^ [s^-1^]^§^ | |
| Sensitivity of motor unbinding | $\lambda_{\text{u,M}}^{*}$ | 2.6×10^-10^ [m] ^§^ | |
| Sensitivity 1 of motor walking | $\lambda_{\text{w,1}}^{*}$ | 1.3×10^-8^ [m] | |
| Sensitivity 2 of motor walking | $\lambda_{\text{w,2}}^{*}$ | 2.2×10^-9^ [m] | |
| Time constant 1 of motor walking | *d*_w,1_ | 1.1×10^-3^ [s] | |
| Time constant 2 of motor walking | *d*_w,2_ | 8.0×10^-2^ [s] | |
| Time constant 3 of motor walking | *d*_w,3_ | 5.9×10^-3^ [s] | |

1. Ferrer JM, Lee H, Chen J, Pelz B, Nakamura F, et al. (2008) Measuring molecular rupture forces between single actin filaments and actin-binding proteins. Proc Natl Acad Sci U S A 105: 9221-9226.

2. Guo B, Guilford WH (2006) Mechanics of actomyosin bonds in different nucleotide states are tuned to muscle contraction. Proc Natl Acad Sci U S A 103: 9844-9849.

3. Uemura S, Higuchi H, Olivares AO, De La Cruz EM, Ishiwata Si (2004) Mechanochemical coupling of two substeps in a single myosin V motor. Nat Struct Mol Biol 11: 877-883.
